# Airway resistance at maximum inhalation as a marker of asthma and airway hyperresponsiveness

**DOI:** 10.1186/1465-9921-12-96

**Published:** 2011-07-15

**Authors:** Nancy T Mendonça, Jennifer Kenyon, Adam S LaPrad, Sohera N Syeda, George T O'Connor, Kenneth R Lutchen

**Affiliations:** 1 Department of Biomedical Engineering, 44 Cummington St., Boston University, Boston, MA 02215, USA; 2 Pulmonary Center, Boston University School of Medicine, 72 E. Concord St., Boston, MA 02118, USA

## Abstract

**Background:**

Asthmatics exhibit reduced airway dilation at maximal inspiration, likely due to structural differences in airway walls and/or functional differences in airway smooth muscle, factors that may also increase airway responsiveness to bronchoconstricting stimuli. The goal of this study was to test the hypothesis that the minimal airway resistance achievable during a maximal inspiration (R_min_) is abnormally elevated in subjects with airway hyperresponsiveness.

**Methods:**

The R_min _was measured in 34 nonasthmatic and 35 asthmatic subjects using forced oscillations at 8 Hz. R_min _and spirometric indices were measured before and after bronchodilation (albuterol) and bronchoconstriction (methacholine). A preliminary study of 84 healthy subjects first established height dependence of baseline R_min _values.

**Results:**

Asthmatics had a higher baseline R_min _% predicted than nonasthmatic subjects (134 ± 33 vs. 109 ± 19 % predicted, p = 0.0004). Sensitivity-specificity analysis using receiver operating characteristic curves indicated that baseline R_min _was able to identify subjects with airway hyperresponsiveness (PC_20 _< 16 mg/mL) better than most spirometric indices (Area under curve = 0.85, 0.78, and 0.87 for R_min _% predicted, FEV_1 _% predicted, and FEF_25-75 _% predicted, respectively). Also, 80% of the subjects with baseline R_min _< 100% predicted did not have airway hyperresponsiveness while 100% of subjects with R_min _> 145% predicted had hyperresponsive airways, regardless of clinical classification as asthmatic or nonasthmatic.

**Conclusions:**

These findings suggest that baseline R_min_, a measurement that is easier to perform than spirometry, performs as well as or better than standard spirometric indices in distinguishing subjects with airway hyperresponsiveness from those without hyperresponsive airways. The relationship of baseline R_min _to asthma and airway hyperresponsiveness likely reflects a causal relation between conditions that stiffen airway walls and hyperresponsiveness. In conjunction with symptom history, R_min _could provide a clinically useful tool for assessing asthma and monitoring response to treatment.

## Background

Structural alterations in asthma include inflammation, increased airway smooth muscle mass, and increased airway wall thickening [1]. These are not easily assessed in patients, so clinicians rely on functional measurements such as spirometry and tests of airway hyperresponsiveness to assess the presence and control of asthma. Another characteristic of asthma is higher airway resistance at maximal inspiration compared to nonasthmatics. Jensen and co-workers [2] used the minimum resistance achieved at maximum inspiration (Rmin) as representing the maximum airway dilation achievable (averaged over the entire lung) by a subject. They showed that Rmin was abnormally high (i.e., less ability to dilate the airway tree) in asthmatic versus nonasthmatic subjects [2]. Salome and co-workers confirmed the reduced ability of asthmatics to dilate after deep inspiration and also showed that the magnitude of dilation was negatively correlated with re-narrowing in nonasthmatics [3]. Black and co-workers [4] showed that respiratory system resistance (R_rs_) measured noninvasively by forced oscillation at maximal inspiration represented the same Rmin as in the Jensen study because the chest wall does not contribute to R_rs _at maximum inspiration. These studies attributed the reduced dilation seen in asthmatics at maximum inspiration to increased stiffness of airway smooth muscle (ASM), reflecting structural characteristics such as hypertrophy and a more contractile state of ASM that may be associated with airway hyperresponsiveness, which is a defining characteristic of asthma.

The goal of this study was to test the hypothesis that the minimal airway resistance achievable during a maximal inspiration (R_min_) is abnormally elevated in subjects with airway hyperresponsiveness. To test this hypothesis, we measured R_rs _in nonasthmatic and asthmatic adults during tidal breathing and at maximal inspiration at baseline, following albuterol-induced bronchodilation, and following methacholine-induced bronchoconstriction. Because airway resistance is related to height, we examined the relationship of R_min _to height in nonasthmatic volunteers so that R_min _could be analyzed as a percent of the predicted value. In addition, we compared R_min _to spirometric indices in terms of their relationship to methacholine airway responsiveness. If R_min _measured by forced oscillation accurately reflects airway hyperresponsiveness and structural abnormalities associated with airflow limitation, it may provide a valuable clinical test to help assess the presence and control of asthma that is easier to perform than spirometry.

## Methods

### Subjects

Participants were recruited by advertisement. Asthmatic participants (n = 35) had a clinical diagnosis of asthma and were taking inhaled bronchodilator. Nonasthmatic subjects (n = 34) denied any history of respiratory symptoms or diagnoses. Participants in both groups were required to have less than 10 pack-years of tobacco smoking. In a substudy to determine the height dependence of resistance measurements, we recruited 84 additional nonasthmatic participants who denied smoking, occupational exposure to smoke or dust, respiratory symptoms, and any respiratory disease history. All subjects provided informed consent, and this research was conducted in compliance with the Helsinki Declaration. This study was approved by Boston University Medical Center IRB, Protocol H-25546, and Boston University Charles River Campus IRB, File 1765E.

### Experimental Protocol

Asthmatic subjects withheld short- and long-acting bronchodilators 6 and 24 hours, respectively, prior to study visits. All subjects attended two test days at least 24 hours apart. On day 1, the forced oscillation system described below was used to measure end-inspiratory R_rs _during tidal breathing and R_min _at maximum inspiration. Subjects took six tidal breaths followed by a slow maximum inspiration followed by a passive exhalation and six more tidal breaths. The procedure was repeated. Subjects then performed spirometry. After baseline studies, subjects inhaled two inhalations of albuterol metered-dose inhaler 90 μg/inhalation via spacer. Forced oscillation and spirometry measurements were repeated after 10 minutes.

On day 2, baseline measurements of R_min _and spirometry were obtained, followed by methacholine challenge. Methacholine (Provocholine^® ^, Methapharm, Canada) was administered in the following concentrations: 0.098, 0.195, 0.391, 0.781, 1.563, 3.215, 6.25, 12.5, and 25 mg/ml. Other than this concentration schedule, testing was performed in accordance with current ATS recommendations using the 5-breath dosimeter protocol [5] using equipment described below. At the conclusion of the challenge (i.e. when a 20% decline in FEV1 occurred or after the final dose of 25 mg/ml, whichever came first), R_min _was measured again, and then 2 inhalations of albuterol were administered. Spirometry and R_min _measurements were repeated 10 minutes after albuterol administration.

In the sample of nonasthmatics studied to establish the relationship of R_min _to height, only R_min _and height were measured.

### Measurement of R_rs_

We measured R_rs _as previously described [4]. Briefly, a 12-in diameter subwoofer delivers an 8 Hz oscillation, with amplitude of ± 1 cmH_2_0, superimposed on spontaneous breathing. Jensen and co-workers [2] showed that because soft-tissue is viscoelastic, it has a tissue resistance that decreases hyperbolically with frequency and that by 8 Hz the lung tissue resistance is negligible and the chest-wall tissue resistance is at its minimum. A three-way valve allows the subject to breathe fresh air through a high-inertance tube. Flow at the airway opening is measured by a pneumotachograph (4700 Series, Hans Rudolph, Kansas City, MO) connected to a differential pressure transducer (ATD02AS, SCIREQ, Montreal, QC). Pressure at the airway opening is recorded with a differential pressure transducer (ATD5050, SCIREQ). These pressure and flow signals are transmitted through demodulator circuits and then to a 10 Hz low-pass filter (S/N 980987, SCIREQ). The filtered signals are sampled at 40 Hz and stored digitally by LabView (National Instruments, Austin, TX). Pressure and flow data were separately low- and high-passed filtered using Matlab software (Natick, MA) at a cut-off frequency of 4 Hz. The signals were processed using a recursive least squares algorithm, described previously[2], to estimate R_rs _eight times per second. Minimum airway resistance, R_min_, was derived as R_rs _at maximum inspiration.

The system used in the substudy of nonasthmatic subjects (n = 84) conducted to determine normative predicted values for R_min _differed only in its differential pressure transducers (Model LCRV, CELESCO, Chatsworth, CA) and had a 1% error from the system used in the main study.

### Spirometry and methacholine challenge methods

Spirometry measurements were made with an integrated spirometer-dosimeter system (KoKoDigidoser^® ^spirometer, Ferraris Respiratory, Louisville, CO) using the DeVilbiss 646 nebulizer. Our measured nebulizer output was 8.7 ± 0.8 uL/breath (mean ± SE), very close to that reported in the literature for this equipment[6]. Predicted values for spirometric indices were based on published regression equations [7]. Spirometry was performed in accordance with published standards [8]. For methacholine challenges, interpolation was used to calculate the provocative concentration causing a 20% drop in FEV_1 _(PC_20_). We also calculated the methacholine dose-response slope [9] as a two-point slope of a line connecting the first and last point of the dose-response curve, measured in units of % decline from baseline FEV_1 _per mg/mL of methacholine, an approach that permits analysis of methacholine responsiveness as a continuous measure even in subjects not experiencing a 20% decline in FEV_1_. For logarithmic transformation of dose-response slope prior to graphic display and correlation analysis, the constant 0.1 was first added to deal with zero or slightly negative values.

### Data Analysis

Among subjects that denied asthma, those with a PC_20 _greater than 25 mg/mL were defined as "nonasthmatic methacholine nonresponders." Among subjects that reported asthma, those with a PC_20 _≤25 mg/mL were defined as "asthmatic methacholine responders."

A second-order linear regression analysis, using the bisquare method[10] to account for undue influence of outliers, was performed to derive a prediction equation for R_min _based on height, using data from 84 nonasthmatic substudy participants and 26 of the 34 nonasthmatic participants in the full study who had a PC_20 _≥25 mg/ml. Predicted values calculated with this equation were used to derive R_min _% predicted = (measured R_min _/predicted R_min_) * 100.

Statistical comparisons were made using paired or unpaired t-tests, or a Mann-Whitney Rank Sum test if a test of normality or equal variance failed, with a significance level of 0.05. Correlations were examined using the Pearson correlation coefficient. For subjects that did not experience a 20% or greater decline in FEV1 by the highest concentration of 25 mg/mL, we assigned a PC_20 _value of 25 mg/mL so that we could calculate a geometric mean for Table 1 (Subject characteristics).

**Table 1:** Characteristics* of 34 nonasthmatic and 35 asthmatic participants.

	Nonasthmatic (n = 34)	Asthmatic (n = 35)
Sex	22 F/12 M	22 F/13 M
Age (yr)	21 ± 2	21 ± 3
Height (cm)	170 ± 10	168 ± 10
Weight (kg)	65 ± 13	66 ± 12
FEV1 (% predicted)	95 ± 10^†^	88 ± 11
FEV1/FVC (% predicted)	100 ± 7^†^	90 ± 9
PC20 (mg/ml)	19 ± 2^†^ median: 25	1.8 ± 5.2 median: 1.3
≥ 25	26	4
16 - 24.9	1	0
8 - 15.9	4	5
< 8	3	26

* Mean ± standard deviation is shown for continuous variables, except for PC20, which is geometric mean ± standard deviation

^† ^p < 0.05

Receiver operator characteristic (ROC) curves to examine the ability of Rmin % predicted and other parameters to predict airway hyperresponsiveness (defined as a PC_20 _< 16 mg/mL) were created by plotting sensitivity (true positive rate) versus 1-specificity (true negative rate), for each value of the test. The best threshold for any test is that which maximizes sensitivity while minimizing the false positive rate, represented by the left upper most value on the curve. The area under the curve (AUC) represents a measure of test accuracy (AUC of 1.0 indicates perfect prediction; AUC of 0.50 indicates prediction no better than chance) and was calculated via numerical integration.

## Results

### Subject Characteristics

We studied 34 nonasthmatic and 35 asthmatic participants with similar demographic and anthropomorphic characteristics (Table 1). Only two of these subjects (both nonasthmatics) were current tobacco smokers. Asthmatics had lower spirometric indices and greater methacholine responsiveness than nonasthmatics. Among the 34 nonasthmatic subjects, 26 were classified as "nonasthmatic methacholine nonresponders" as defined above. Among the 35 subjects that reported asthma, 31 subjects were classified as "asthmatic methacholine responders" as defined above. The 84 additional nonasthmatic subjects, who underwent only forced oscillation and anthropomorphic measurements, had a mean age of 21 and were 55% males.

### Dynamic Rrs tracings and determination of R_min _in representative subjects

**Figure 1:**
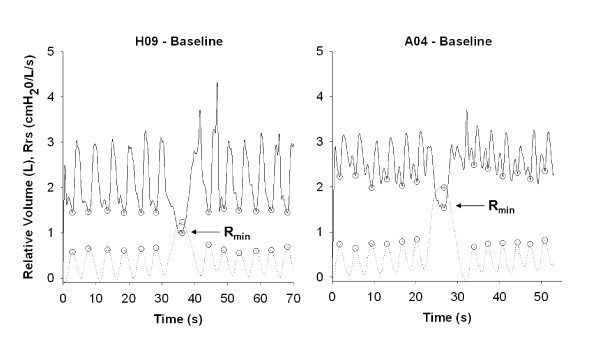
**Typical respiratory system resistance tracings for a nonasthmatic and an asthmatic subject**. Typical trace of respiratory system resistance (R_rs_) at 8 Hz and relative inhaled volume (above functional residual volume) for a nonasthmatic (H09) and asthmatic (A04) subject at baseline. Both participants are female and of similar age, height, and weight. End-inspiration R_rs _values are used in analysis (open circles). The minimum resistance achieved at maximum inspiration is termed R_min_. The R_rs _is plotted as a solid line, and the inhaled volume is plotted as a dotted line.

Typical tracings of R_rs _and relative volume for a nonasthmatic and an asthmatic subject are shown in Figure 1. For the asthmatic participant shown, the mean end-inspiratory pre-deep inspiration R_rs _was 2.36 cmH_2_0/L/s, and R_min _was 1.46 cmH_2_0/L/s, values approximately 50% higher than those of the nonasthmatic subject shown (1.45 and 0.99 cmH_2_0/L/s for R_rs _and R_min_, respectively).

### Relationship Between R_min _and Height

**Figure 2:**
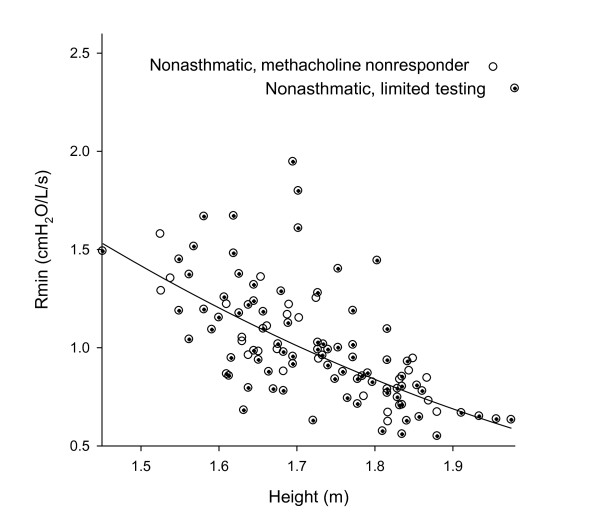
**Plot of R_min _versus height for nonasthmatic subjects**. R_min _(cmH_2_O/L/s) is plotted by height (m) for 100 nonasthmatic subjects, including 84 subjects recruited for limited testing (+) and 26 nonasthmatic methacholine nonresponders (0), as described in the text. The superimposed regression line is derived from a second order linear regression (r^2 ^= 0.60).

We examined the relationship between R_min _and height among the 84 subjects that underwent limited testing plus the 26 nonasthmatic methacholine nonresponders in the full study. These two groups displayed a similar relationship between R_min _and height (Figure 2) and were therefore analyzed together. Regression analysis of these 100 subjects revealed the following relationship:

The R^2 ^for this model (regression line superimposed on Figure 2) was 0.60, indicating a relationship between R_min _and height of similar strength to that between spirometric measurements and height [7]. R_min _was not significantly related to sex or body-mass index after accounting for height.

### R_min _% predicted as an indicator of asthma and airway hyperresponsiveness

**Figure 3:**
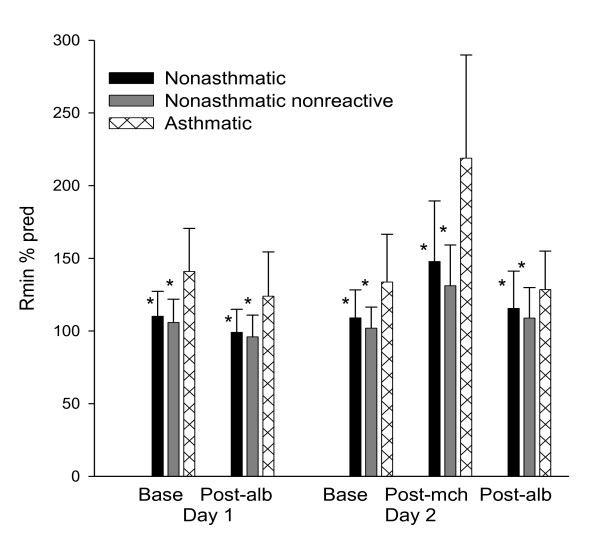
**Plot of R_min _% predicted for asthmatic and nonasthmatic subjects**. R_min _%predicted for all 34 nonasthmatic (black) and 35 asthmatic (hatched) participants as well as the subgroup of 26 nonasthmatic methacholine nonresponders (gray). *indicates significant difference from asthmatic group in each condition (p < 0.05)

The baseline R_rs _(end-inspiration values averaged over 6 pre-deep inspiration tidal breaths), R_min_, and R_min _% predicted differed significantly between asthmatics and nonasthmatics, as did spirometric indices (Table 2). These differences were even more pronounced when comparing nonasthmatic methacholine nonresponders and asthmatic methacholine responders (Table 2). The R_min _% predicted was significantly greater among asthmatics than nonasthmatics in all conditions (baseline, post-albuterol, post-methacholine), differences that were even more pronounced when comparing asthmatics to nonasthmatic methacholine nonresponders (Figure 3). Among subjects without asthma, the R_min _was greater among those with a PC_20 _≤25 mg/mL than among those with a PC_20 _> 25 mg/mL (R_min _% predicted 131.7 +/- 5.3 SE vs. 102.1 +/- 2.9 SE, P < 0.0001).

**Table 2:** Baseline physiologic measurements* in asthmatic and control subjects and in subgroups of these subjects.

		All subjects			Subgroups	
**Physiologic** **measurement**	**Nonasthmatic** **(n = 34)**	**Asthmatic** **(n = 35)**	**P value**	**Nonasthmatic methacholine nonresponders** **(n = 26)**	**Asthmatic methacholine responders** **(n = 31)**	**P value**

FEV1 % predicted	95 ± 10	88 ± 11	0.009	97 **± **8	88 ± 11	0.002

FEV1/FVC % predicted	100 ± 7	90 ± 9	< 0.0001	102 ± 6	90 ± 9	< 0.0001

FEF25-75 % predicted	93 ± 20	69 ± 20	< 0.0001	99 **±**18	68 **±**18	< 0.0001

R_rs_, cmH20/L/s	2.21 ± 0.48	2.91 ± 0.99	0.0006	2.10 **±**0.24	2.95 **±**1.04	0.0005

R_min_, cmH20/L/s	1.12 ± 0.31	1.39 ± 0.41	0.004	1.02 ± 0.24	1.41 ± 0.42	0.0001

R_min_, % predicted	109 ± 19	134 ± 33	0.0004	102 ± 14	137 ± 33	< 0.0001

* Mean ± standard deviation

**Figure 4:**
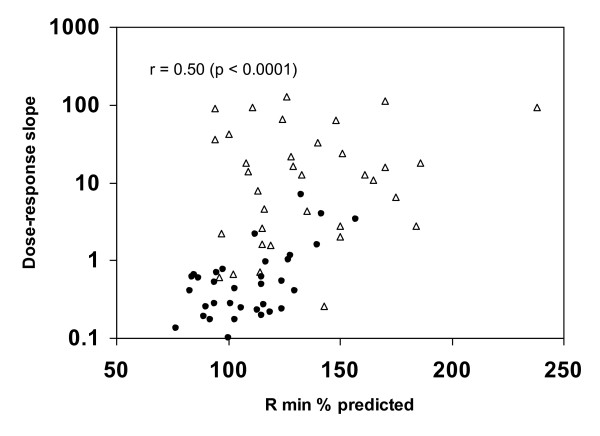
**Plot of methacholine dose-response slope versus R_min _percent predicted**. Scatter plot of dose-response slope versus baseline R_min _% predicted for nonasthmatic (closed circles) and asthmatic (open triangle) participants.

**Figure 5:**
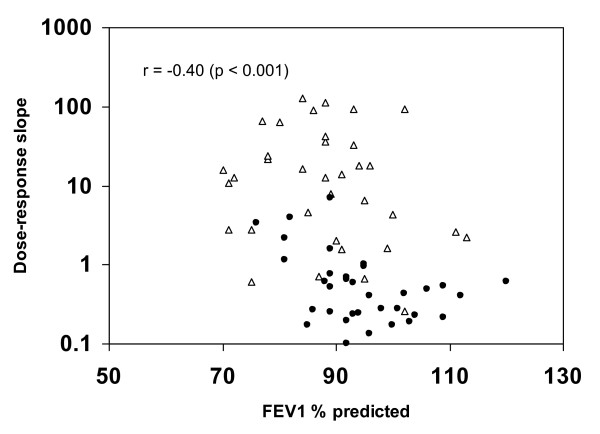
**Plot of methacholine dose-response slope versus FEV_1 _percent predicted**. Scatter plot of dose-response slope versus FEV_1 _% predicted for nonasthmatic (closed circles) and asthmatic (open triangle) participants.

**Figure 6:**
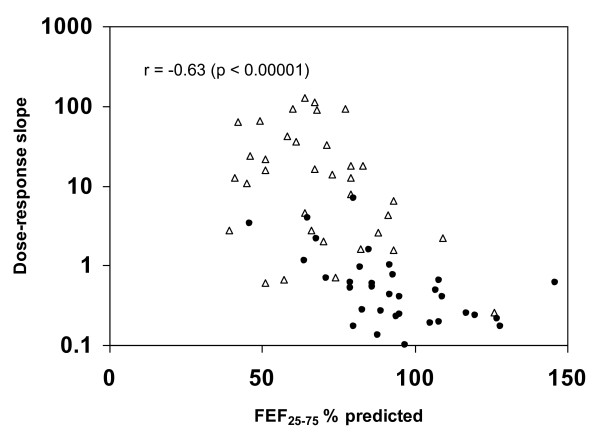
**Plot of methacholine dose-response slope versus FEF_25-75 _percent predicted**. Scatter plot of dose-response slope versus FEF_25-75 _% predicted for nonasthmatic (closed circles) and asthmatic (open triangle) participants.

In Figures 4, 5, 6, the methacholine dose-response slope is plotted versus R_min _% predicted (Figure 4), FEV_1 _% predicted (Figure 5), and FEF_25-75 _% predicted (Figure 6) for nonasthmatic (closed circles) and asthmatic (open triangle) participants. These plots reveal that the log_10 _dose-response slope was significantly correlated with Rmin % predicted (r = 0.50, p < 0.0001), FEV_1 _% predicted (r = -0.40, p < 0.001), and FEF_25-75 _% predicted (r = -0.63, p < 0.00001). Defining airway hyperresponsiveness as a methcholine PC_20 _< 16 mg/mL (corresponding to a dose-response slope > 1.2), 80% of the subjects with baseline R_min _< 100% predicted did not have airway hyperresponsiveness, while 100% of subjects with R_min _> 145% predicted had hyperresponsiveness, regardless of clinical classification as asthmatic or nonasthmatic.

**Figure 7:**
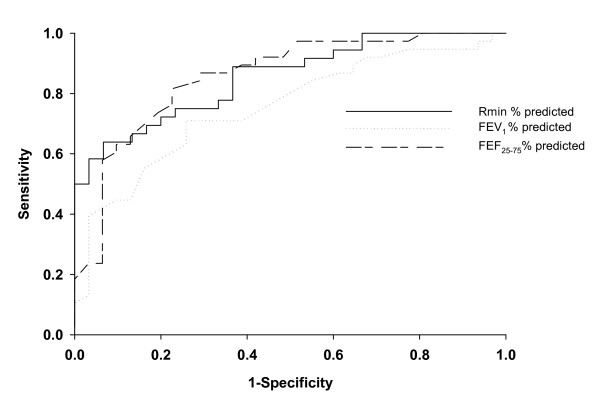
**Receiver operator characteristic curves for R_min_, FEV_1_, and FEF_25-75 _as predictors of airway hyperreactvitiy**. Receiver operator characteristic (ROC) curves for R_min_, FEV_1_, and FEF_25-75 _as predictors of airway hyperresponsiveness (PC_20 _< 16 mg/ml). The thresholds yielding the highest combined sensitivity and specificity were 115, 91, and 82 for R_min _% predicted, FEV_1 _% predicted, and FEF_25-75 _% predicted, respectively. The area under the curve (AUC) was 0.85, 0.78, and 0.87 for R_min _% predicted, FEV_1 _% predicted, and FEF_25-75 _% predicted, respectively.

ROC curves were used to formally compare the ability of these measurements to distinguish hyperresponsive subjects (defined as PC_20 _less than 16 mg/ml) from subjects without hyppresponsiveness and to identify the optimal threshold levels for distinguishing these groups (Figure 7). The thresholds yielding the highest combined sensitivity and specificity were 115 for R_min _% predicted, 91 for FEV_1 _% predicted, and 82 for FEF_25-75 _% predicted. The AUC for R_min_, FEV_1_, and FEF_25-75_, were 0.85, 0.78, and 0.87, respectively. The AUC for both the FEV_1_/FVC ratio and FEF_25-75_/FVC ratio (not shown in figure) was 0.81. The percent increase in FEV_1 _following albuterol administration on the first day of the protocol was also analyzed and was comparable to R_min _% predicted (AUC = 0.85 with a threshold of 3.7% FEV_1 _increase). ROC curves were also calculated for hyperresponsiveness defined as a PC_20 _< 25 mg/ml, and in this case the R_min _% predicted had the highest AUC at 0.87.

## Discussion

Our goal was to test the hypothesis that the minimal airway resistance achievable during a maximal inspiration (R_min_) is abnormally elevated in subjects with airway hyperresponsiveness. The breathing maneuver required to measure R_min _by the forced oscillation method is less burdensome and less subject to performance-related errors than is spirometry. We observed that the baseline R_min_, as a percent predicted value based on height, identifies people with airway hyperresponsiveness approximately as well as FEF_25-75 _and slightly better than FEV_1_.

Previous reports suggested a decreased ability of asthmatic airways to dilate in response to a deep inspiration, a deficiency that was accentuated after bronchial challenge [2,11] Our measurements in a larger sample of subjects agree with these previous observations. At baseline, R_min_, an inverse measure of airway caliber, was significantly higher in asthmatics compared to nonasthmatics. Following inhalation of albuterol, subjects with asthma still had higher R_min _than nonasthmatics (Figure 3). In fact, asthmatic subjects had a higher mean R_min _after albuterol than the nonasthmatic methacholine nonresponder group before albuterol (not shown), indicating that in subjects with asthma, albuterol cannot always dilate airways to levels achievable in nonasthmatic airways. This suggests that either albuterol does not relax the airway smooth muscle of asthmatics to the same extent as nonasthmatics or that the airway walls have become stiff or narrowed by other mechanisms. In that our data on response to albuterol suggest that asthmatics have an approximately similar decline in R_min _in response albuterol as nonasthmatics (reduction in R_min _% predicted 17 ± 6.3 SE vs. 11 ± 2.2 SE for asthmatics and nonasthmatics, respectively; p = 0.38), this may favor the explanation of residual differences in the airway wall independent of ASM tone. It must be noted that the dose of albuterol administered in our protocol, i.e. 180 ug (two inhalations), is not a maximally bronchodilating dose. When the stiffer asthmatic airway is constricted by methacholine, the inability to dilate with a deep inspiration is exaggerated compared to nonasthmatic participants, the R_min _% predicted increasing in response to methacholine by 85 ± 12 SE vs. 38 ± 5.6 SE (p < 0.001) in asthmatics and nonasthmatics, respectively (Figure 3).

There are several factors that influence airway caliber, including airway smooth muscle tone and stiffness, the passive properties of the airway wall (e.g. airway wall thickening), parenchymal tethering and transmural pressure acting to distend the airway. Several of these can be influenced by airway wall remodeling. Direct measurement of airway distensibility in the intact lung (i.e. the relationship between airway caliber and airway distending pressure) is difficult. Recent work by Brown et al. confirms the ability to indirectly assess airway distensibility non-invasively using forced oscillations [12,13]. Specifically, distensibility was quantified as the linear slope of respiratory system conductance (1/R_rs_) and volume between 75% and 100% of total lung capacity. This slope was decreased in asthmatics and unaffected by reduction of bronchomotor tone with albuterol. Brown et al. concluded that reduced airway distensibility in asthmatics is consistent with structural changes associated with airway wall remodeling and is not reflective of increased airway smooth muscle tone. This is consistent with the data of our study. Another key determinant of the ability to dilate could be lung elastic recoil pressure; past studies have reported a significant loss of recoil in moderate-to-severe though perhaps not mild asthma[14-16]. We did not measure elastic recoil in our study and can only speculate on its role.

Several limitations of our study must be recognized. The sample size was relatively small (n = 69 for the full protocol and n = 84 for the limited testing to establish predicted values for Rmin), the age range was limited to to 18-29 years, and most subjects were Caucasian race. A larger and more diverse sample would permit better evaluation of the potential relationship of R_min _to age and race, as well as subgroup analyses. In addition, the asthmatic subjects had mild to moderate disease, so the full spectrum of asthma was not reflected in our sample, and we were not able to assess the correlation of R_min _with clinical status. It is possible that there could be important differences in the physiology of milder versus more severe asthma. Finally, the deep inhalations performed during the dosimeter protocol for methacholine challenge have been reported to result in bronchoprotection and falsely negative challenge results among mild asthmatics, compared to the tidal breathing protocol[17,18]. It would be of interest to have data on the relationship of R_min _to airway responsiveness assessed by both protocols.

## Conclusions

Our study reveals that after adjusting for height, R_min _differs between asthmatics and nonasthmatics, predicts methacholine responsiveness, increases with administration of methacholine, and decreases with albuterol. Compared to spirometry, this test requires less patient effort and is easier for a technician or clinic staff member to administer with technically acceptable results. In conjunction with symptom history, R_min _could provide a clinically useful tool for assessing asthma control and monitoring the response to treatment. Longitudinal studies are needed to assess the utility of R_min _as an indicator of asthma control and response to asthma therapy.

## List of abbreviations

AUC: area under the curve; PC_20 _:provocative concentration causing a 20% drop in FEV_1; _ROC: receiver operator characteristic; R_min_: minimal airway resistance achievable during a deep inspiration; R_rs_: respiratory system resistance

## Competing interests

The authors declare that they have no competing interests.

## Authors' contributions

NTM contributed to study design, acquisition of data, analysis and interpretation of data, and drafting and revising the manuscript. JK contributed to study design, acquisition of data, analysis and interpretation of data, and drafting and revising the manuscript. ASL contributed to acquisition of data, analysis and interpretation of data, and drafting and revising the manuscript. SNS contributed to study design, acquisition of data, analysis and interpretation of data, and drafting and revising the manuscript. GTO contributed to study design, acquisition of data, analysis and interpretation of data, and drafting and revising the manuscript. KRL contributed to study design, acquisition of data, analysis and interpretation of data, and drafting and revising the manuscript. All authors read and approved the final manuscript.
